# Circadian rhythms as modulators of brain health during development and throughout aging

**DOI:** 10.3389/fncir.2022.1059229

**Published:** 2023-01-19

**Authors:** Rachel Van Drunen, Kristin Eckel-Mahan

**Affiliations:** Institute of Molecular Medicine, The University of Texas Health Science Center at Houston, Houston, TX, United States

**Keywords:** circadian, suprachiasmatic nucleus, memory, aging, chronotherapy, synaptic plasticity

## Abstract

The circadian clock plays a prominent role in neurons during development and throughout aging. This review covers topics pertinent to the role of 24-h rhythms in neuronal development and function, and their tendency to decline with aging. Pharmacological or behavioral modification that augment the function of our internal clock may be central to decline of cognitive disease and to future chronotherapy for aging-related diseases of the central nervous system.

## Introduction

Before humans are even born, signals, and hormones in the womb prime the fetus for the diurnally changing environment of the outside world. Early within the first year of birth, activity and sleep cycles synchronize to the 24-h rotation of the Earth on its axis. Research within the past few decades reveals these internal, ubiquitous, biological cellular clocks can shape some of the most important aspects of central nervous system development in humans. Neuronal connectivity, characterized by synaptic connections, dendritic spines, and axonal projections to name a few, is integral to our cognitive functions and daily behaviors. When these attributes of neuronal connectivity are disrupted, dysregulated, or simply deteriorate over time, a variety of cognitive defects can arise, including defects in learning and memory and behavioral abnormalities like anxiety and depression. Aging, which is associated with a decline in the robustness of the internal circadian clock, also leads to several neurological disorders such as Alzheimer’s disease (AD). This review will discuss some of the research linking the circadian clock system and neural plasticity from birth to death.

**FIGURE 1:**
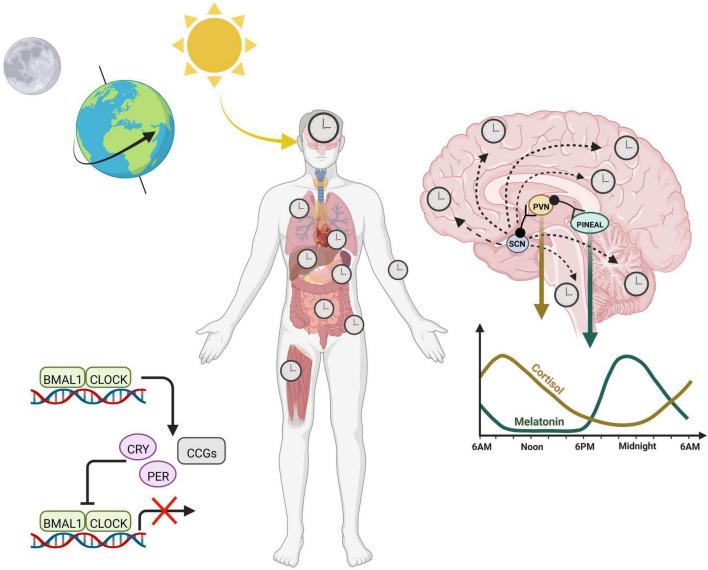
Circadian rhythms across the body are entrained by the 24-h environment. Diurnal light exposure entrains the “master clock”, known as the suprachiasmatic nucleus (SCN). The SCN coordinates daily rhythms in the brain as well as the periphery *via* a combination of neuronal signals and hormones, including cortisol and melatonin, which fluctuate opposite to each other and promote sleep/wake cycles. At the cellular level, the core transcription translational feedback loop in the nucleus is characterized by BMAL1 and CLOCK binding to E-box containing genes to regulate transcription of numerous clock controlled genes (CCGs) including their own repressors, PERIOD (PER) and CRYPTOCHROME (CRY). Heterodimers PER and CRY translocate back into the nucleus to inhibit the CLOCK:BMAL1 complex, thereby inhibiting their own production.

A transcriptional-translational feedback loop (TTFL) serves as the core circadian mechanism from which diurnal cellular rhythms arise. Known as the core loop, this negative feedback mechanism consists of the transcription factors, circadian locomotor output cycles kaput (CLOCK) and brain and muscle ARNT-like protein (BMAL1), which heterodimerize in the nucleus to promote the expression of numerous E-box containing genes. Among these target genes, are the period (PER) and cryptochrome (CRY) repressors, which upon translation in the cytoplasm, will translocate back into the nucleus to function as direct repressors of the CLOCK:BMAL1 transcriptional heterodimer (Partch et al., 2014). CLOCK:BMAL1 activity resumes only when the PER/CRY complex is primed for degradation by additional regulators, such as the serine/threonine kinase casein kinases (CK1δ and CK1ε), which phosphorylate PER, initiating its ubiquitination (Reppert and Weaver, 2002). CRY is phosphorylated by the metabolic sensor AMP activated protein kinase (AMPK), which marks it for proteasomal degradation (Lamia et al., 2009). This negative feedback loop, present in nearly all mammalian cells, mediates rhythmic expression of hundreds of clock-controlled genes (Figure 1).

At a grander scale, the tissue clocks of the body are synchronized by external cues from the environment, otherwise known as *Zeitgebers* (or “time-givers”). Light, a potent *zeitgeber*, is a particularly strong entrainer of the brain, and activates the suprachiasmatic nucleus (SCN) *via* light sensitive retinal ganglion cells in the eye. Thus, the SCN entrains to the day/night cycle, and helps synchronize peripheral clocks (Hastings et al., 2003). Organization of peripheral rhythms is carried out by both direct neuronal projections as well as the release of neuroendocrine hormones, which can be circulated in an endocrine fashion. Melatonin is produced by the pineal gland and at nighttime, reaches its diurnal zenith, stimulating the onset of sleep. On the other hand, cortisol surges in the morning, transitioning the body into wakefulness before steadily decreasing to reach its trough at bedtime. While light acting through the SCN entrains these hormones to rise and fall at certain times, the absence of light does not exterminate these rhythms based on the feedback mechanism of the cellular clock. This intrinsic aspect of our internal clock means that even in constant darkness, internal clocks maintain time, which is referred to as “free-running” of the endogenous clock. Thus, circadian rhythms are endogenous and self-perpetuating.

Studies over the last two decades have revealed that decoupling of the central clocks in the brain from the peripheral tissue clocks leads to desynchrony between clocks and an increased risk for developing certain diseases including obesity, diabetes, and even Alzheimer’s disease (AD) (Parsons et al., 2015; Bokenberger et al., 2018; van der Vinne et al., 2020). Chronic exposure to environmental stressors such as jet lag, high fat diet, and shift work leads to chronodisruption, increasing an individual’s risk for long term health problems (Cho et al., 2000; Drake et al., 2004; Parsons et al., 2015; Bokenberger et al., 2018; van der Vinne et al., 2020). Here, we focus on how the circadian system shapes our internal neural pathways throughout lifespan, highlighting reasons why circadian disruption might increase disorders of the central nervous system associated with aging.

## Circadian rhythms and neural plasticity in development

The evolution of circadian rhythms during development is important for the long-term health of the organism. Failure of circadian rhythms to arise or perturbation of the clock during development can negatively impact specific biological functions and behaviors. The importance of circadian rhythms during development has been demonstrated in both humans and pre-clinical models. For example, one study analyzing data from almost 200000 women engaged in shift work during pregnancy revealed that chronodisruption during pregnancy is associated with preeclampsia, gestational hypertension, pre-term delivery, and small infants for the corresponding gestational age (Cai et al., 2019). Studies of pregnant rats reveal that disruption of circadian rhythms during fetal development is associated with increased risk for several physiological problems including hypertension (Tain et al., 2017). Moreover, studies in preclinical rodent models suggest neurological problems, such as mood disorders, may be related to circadian perturbation of the fetus during pregnancy (Zhang et al., 2017). It is not yet clear the extent to which the circadian system affects neurological development in humans, though studies in preclinical mammalian models provide evidence of cause and effect relationships. This section will discuss the current knowledge pertaining to the role of the circadian system in neural plasticity during development.

### Circadian system development pre- and postnatally

Known as the “Developmental Origin of Health and Disease” (DOHaD) theory, growing evidence supports the idea that lifestyle-related diseases are actually formed during the developmental stages of an organism, including fertilization, and embryonic through neonatal stages (Heindel and Vandenberg, 2015). Though commonly recognized causes of disease predisposition include nutrition, stress, or environmental chemicals, data suggests that circadian disruption should be added to this list. Research over the past decade indicates the circadian system begins developing prenatally *via* entrainment from the mother. SCN receptors for melatonin are reportedly expressed as early as 18 weeks of age in humans followed by dopamine receptor expression at 22 weeks of age (Reppert et al., 1988). Maternal hormones including melatonin and dopamine pass the placenta and the fetal blood brain barrier to stimulate the fetus (Okatani et al., 1998). Data suggests that maternal rhythms are important to brain development and circadian rhythms in the offspring. For example, progeny of pregnant primates housed under constant light exposure have suppressed postnatal melatonin and body temperature rhythms (Serón-Ferr et al., 2013). Similar light disruption experiments in pregnant rats showed that progeny ultimately showed reduced rhythms in circadian gene expression as well as NMDA receptor gene expression in the hippocampus at adulthood (Voiculescu et al., 2016). Strikingly, affected rat pups showed permanent cognitive defects into adulthood, performing more poorly on spatial memory tests than pups whose mothers were not subjected to constant light.

**FIGURE 2:**
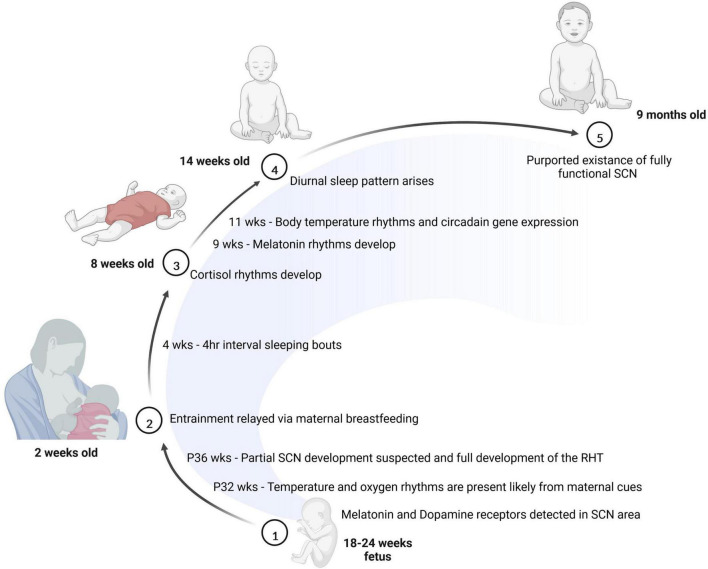
The development of the circadian system is initiated prior to birth. Between 18 and 24 weeks of age, melatonin and dopamine receptors are detectable in the SCN. Soon after birth, many entrainment cues are passed to the baby through breast milk. At 8 weeks of age, cortisol rhythms have emerged in the infant, and by 14 weeks of age, most babies show a diurnal sleep pattern. Finally, at 9 months of age, the baby is thought to have a fully functional SCN.

Other factors, including maternal body temperature, breastfeeding and hormone release also contribute to entrainment of the fetus (Reppert, 1995). The emergence of the sleep-wake cycle is closely tied to the production of two key hormones: cortisol and melatonin. While cortisol is involved in wakefulness and arousal, melatonin is involved in sleep onset. Prenatal infants receive these hormones from the mother, and begin producing cortisol as early as 8 weeks of gestation; however, they do not produce the melatonin until after birth (Kennaway et al., 1992; Johnston et al., 2018). Interestingly, depending on the season the amount of melatonin produced by infants may vary (Sivan et al., 2001). Newborns receive melatonin and cortisol *via* the breast milk of the mother where the level of hormones in the breast milk varies throughout the day. It is not exactly clear the extent to which these fluctuations in the breastmilk affect the circadian timing of infants, but some studies suggest that these rhythms may be important for consolidated sleep after birth (Cubero et al., 2005; Engler et al., 2012; Doan et al., 2014). For example, infants breastfed at nighttime reportedly have higher sleep efficiency with less fragmentation and longer sleep duration compared to infants fed formula at nighttime (Engler et al., 2012; Doan et al., 2014). Moreover, as early as 2 weeks of age, infants have been found to produce rhythmic cortisol, which may correspond to the shift from sleeping intermittently throughout the day to sleeping at approximately 4 h intervals (Anders and Keener, 1985; Santiago et al., 1996). At 5 weeks of age, an infradian rhythm of sleep arises and at approximately 9 weeks of age, rhythmic melatonin production begins (Kleitman and Engelmann, 1953; Rivkees, 2003). By 11 weeks of age in human infants, a diurnally oscillating body temperature along with rhythmic circadian gene expression emerges. At 15 weeks, the sleep-wake cycle of infants follows a more apparent trend (Kleitman and Engelmann, 1953), and by 6–9 months of age, infant generally sleep through the night (Jenni and Carskadon, 2004). One study looking at infants of approximately 6.3 months of age revealed that by this time individual functional connectivity based on prolonged EEG recordings was relatively stable during the sleep and wake cycles (Smith et al., 2021). The researchers found the infants’ network connectivity appeared to be entrained to a 24-h cycle and modulated by their state of consciousness. This reveals that even at an infantile stage, neural connectivity follows a circadian pattern. Figure 2 summarizes what is known regarding the timeline for circadian rhythm development in humans.

### Sleep disruption in infants

Studies suggest that sleep issues arising from the disruption of the circadian system can lead to improper formation of neural connections from which behavioral phenotypes arise. For example, several studies carried out in children have revealed a relationship between sleep impairments and behavioral/mood problems including aggressive behavior, attention, anxiety, and hyperactivity (Gregory and O’Connor, 2002; Hoedlmoser et al., 2010; Kamphuis et al., 2014). In a study carried out on school aged children with sleep deficits, researchers found significant correlations between sleep quality and a child’s ability to carry out cognitive tasks (Sadeh et al., 2002). Preclinical rodent studies also support the association between sleep disruption in early life and impaired cognition that is manifest sometimes much later in life. For example, mouse pups placed under rapidly changing light/dark cycles until weaning (21 days), display impaired spatial and problem-solving tasks as well as increased anxiety-like behavior and decreased neuronal complexity in the prefrontal cortex (Ameen et al., 2022). In another study, researchers exposed mice to ambient light *via* an 8:8-h light/dark cycle from embryonic day one to postnatal day 42. This abnormal light paradigm resulted in mice that expressed an autistic-like phenotype in adulthood (Fang et al., 2021). This tie to autism or autism spectrum disorder (ASD) is present in humans as well. ASD is often comorbid with sleep disorders (Kotagal and Broomall, 2012) where sleep problems are reported in 43–88% of children with ASD (Richdale, 1999). Moreover, approximately 65% of the ASD individuals surveyed in one study had reduced levels of melatonin (Melke et al., 2008). While most studies linking ASD to sleep and circadian disruption are association based, genetic studies have begun to uncover mutations or polymorphisms in clock genes of individuals screened for ASD. One such genetic study screening of 110 ASD individuals and their parents suggested an association between single-nucleotide polymorphisms in *Per1* and *Npas2* and ASD (Nicholas et al., 2007). While the mechanistic basis for this connection is still an enigma, these studies point to a connection between circadian rhythms in development, sleep and ASD that needs to be further studied.

## SCN development

The function of the circadian clock in the developing brain has been studied extensively, though certain aspects still remain enigmatic. Rodent models provide evidence that although clock genes are expressed early in development, rhythmic expression of clock genes and metabolic rhythms occurs much later [reviewed in Landgraf et al. (2014a)]. In humans, the SCN of the fetus is identifiable between the end of the second trimester and the beginning of the third trimester. Additional studies in a variety of organisms support the SCN-dependent entrainment of the fetus during gestation (Reppert and Schwartz, 1983, 1984; Schwartz et al., 1983; Weaver and Reppert, 1987). Though the SCN synchronizing neuropeptides vasopressin (AVP) and vasoactive intestinal polypeptide (VIP) are expressed at varying stages of embryonic development in rodent models, in humans they are detected at the 27th (AVP) and 31st (VIP) days of pregnancy, both increasingly expressed in early life after birth.

The SCN originates with part of the neurepithelium and throughout development specific genes characterize SCN development. These include six homeobox 3 (six 3), Lim homeodomain transcription factor 1 (Lim 1) and RAR-related orphan receptor alpha (Rora) (VanDunk et al., 2011). Though the SCN structure emerges relatively early in development, even before birth, its capacity to function as a synchronized pacemaker, capable of controlling circadian physiology occurs at later stages of development. This is probably due in part to the relatively late emergence of synchronizing neuropeptides such as AVP, which while detectable as early as the 27th week of pregnancy in human embryonic hypothalamic tissue, continues to increase in expression throughout the first year after birth (Swaab et al., 1990). A similar increase in VIP neurons during the first few years after birth has also been documented (Swaab et al., 1994). A recent “omics” study addressing the emergence of fetal SCN rhythms prior to the induction of the canonical clock showed that early rhythms in genes involved in neurodevelopment and cell-cell communication could be observed prior to consolidated clock gene rhythms (Greiner et al., 2022). Fetal rhythms were highly dependent on the rhythmicity of the pregnant rat, indicating that rhythmic maternal cues are important for the development of fetal SCN rhythmicity. Though limited studies have addressed the temporal development of SCN efferent projections, one study suggests that efferent growth may begin around P1 in hamster pups, with a large increase in the density of SCN efferents to known target sites occurring several days after birth (Muller and Torrealba, 1998).

In humans, preterm infants demonstrate rhythmicity in body temperature between 29 and 35 weeks or age (Mirmiran and Kok, 1991), suggesting that body-wide rhythms may emerge during pregnancy in advance of this time period. Though temperature rhythms usually appear first in an infant, some studies indicate that circadian rhythms in the sleep/wake cycle appear shortly thereafter, at around 45 days of age, concomitant with the time at which melatonin begins to sync with sunset (McGraw et al., 1999). This relatively early onset of rhythms in infants is probably attributable to both normal exposure to natural light, as well as regular social cues.

## Rhythms in plasticity

**FIGURE 3:**
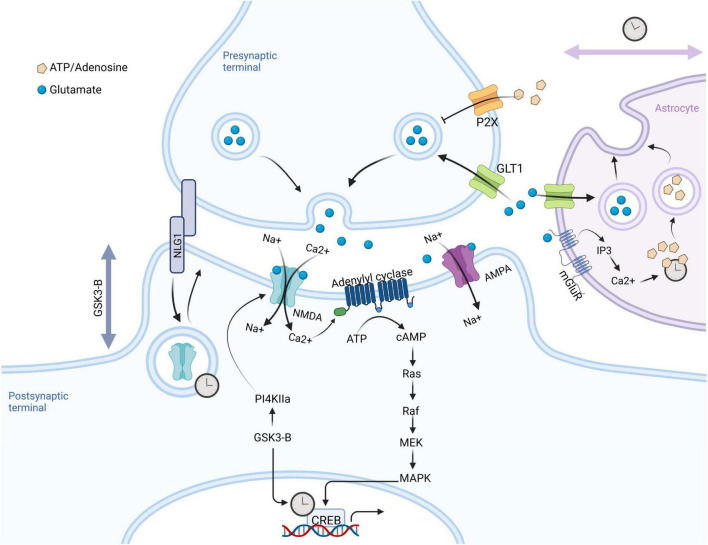
Synaptic mechanisms under circadian control. Glutamate released from the presynaptic terminal binds to NMDA and AMPA receptors stimulating an influx of Na^+^ and Ca^2+^, respectively. Ca^2+^- activated adenylyl cyclases lead to increased cAMP, ultimately resulting in CREB phosphorylation and activation of gene transcription. This pathway is both light-responsive in the SCN and under circadian control in the hippocampus, where it regulates learning and memory. Meanwhile the clock-controlled gene, GSK3β, regulates post-synaptic dendritic growth. Astrocytes play an important role in circadian timing in the SCN, where their circadian nighttime activity suppresses activity of SCN neurons by regulation of extrasynaptic glutamate levels. Intracellular calcium levels in astrocytes are also highly dynamic over the 24-h cycle.

The circadian system is an important factor in neural plasticity in the adult brain, which includes the cellular and molecular changes in the nervous system that result in regeneration and adaptation to the environment, which are ultimately manifest at the level of memory and learning and behavior. By modulating changes in hormone production, the sleep-wake cycle, and neurotransmitters, the circadian system is able to influence neural connections. When the circadian clock is ablated, neurological and behavioral abnormalities arise, revealing the widespread role of the circadian system in the brain. For example, Macaques lacking *Bmal1* show reduced sleep and increased activity during the sleep phase, as well as symptoms of anxiety and depression (Qiu et al., 2019). As numerous synaptic molecules have oscillatory expression or activity, it is not surprising that diurnal rhythms in plasticity can affect cognitive functions like learning, memory, and mood. For example, SCN P/Q- and T-type voltage-dependent calcium channels are circadian in expression (Nahm et al., 2005), and SCN neurons show circadian rhythms in intracellular calcium concentrations (Colwell, 2000). Interestingly, while over 1,200 daily oscillations have been observed in the mammalian hippocampal transcriptome under normal conditions, a rodent model of epilepsy reveals even more daily transcriptomal variation in the hippocampus, with little overlap in oscillating events compared to control hippocampus (Debski et al., 2020). In this particular study, diurnal oscillation of the oxidative phosphorylation pathway was present in the normal hippocampus, but was absent in the mouse model of epilepsy. Adenylyl cyclase activation and downstream MAPK activity, two processes heavily involved in hippocampus-dependent memory consolidation in the hippocampus are highly rhythmic (Eckel-Mahan et al., 2008). In neurons, MAPK activity leads to activation of the transcription factor CREB (Impey et al., 1998), a transcription factor that is both light-inducible and highly rhythmic in both the hippocampus and the SCN (Obrietan et al., 1998, 1999). These studies serve as only a few of many examples that pathways involved in neuronal activity and plasticity are highly rhythmic in the brain, and might be exploited from the context of disease diagnosis and drug treatment. Figure 3 depicts several of the molecular events known to be under control in neurons, and which may eventually serve as targets for the purposes of chronotherapy.

### Circadian rhythms and plasticity in flies

Much information regarding the role of our 24-h oscillations in neural plasticity has been accomplished by research conducted using non-vertebrate models. Insects have a considerably simplified nervous system and thus have been key to unlocking the changes in neural growth. As plasticity is often measured by changes in neuron morphology, neural connectivity, and neuron generation, *Drosophila melanogaster* have been a useful model in which to study and measure plasticity.

The *D. melanogaster* nervous system is composed of approximately 150 clock neurons, which are organized to both synchronize and maintain circadian rhythms in the organism. The fruit fly brain is divided into seven major groups based on the anatomical locations. There are three groups of dorsal neurons (DN, DN1-3) and four groups of lateral neurons (LNds, LPNs, 1-LNvs, and s-LNvs). The clock neuron clusters produce and diurnally release the hormone pigment dispersing factor (PDF), which is key for internal synchronization and clock output pathways (Renn et al., 1999). Altogether, the pacemaker neurons regulate circadian rhythms in the brain and body of the fly *via* hormonal and neural circuit connections. Pacemaker neurons use leucokinin neuropeptide (LK) and its receptor (LK-R) circuit to entrain locomotor rhythms and sleep in flies (Cavey et al., 2016). The dorsal terminals of the s-LNvs alter their complexity throughout the day, having the highest arborization in the morning (Fernandez et al., 2008). At nighttime, axon terminals of the s-LNv retract in an actin and myosin-mediated mechanism, which is mediated by the circadian-regulated Rho1 protein (Petsakou et al., 2015). Using the GFP reconstitution across synaptic partners (GRASP) technique to monitor synapse connectivity in the s-LNvs revealed that these neurons can engage in different synaptic connections throughout the day (Gorostiza et al., 2014). These diurnal changes in complexity include a change in the number of synapses across 24-h. For example, the number of motor neuron synapses in fruit flies changes in both LD and DD conditions (Ruiz et al., 2013). Moreover, optogenetic silencing experiments have revealed that the LNv-DN1a circuit is responsible for time of day dependent light responsiveness whereby DN1a neurons are remodeled at an antiphase time to the remodeling of LNv neurons (Song et al., 2021). Although the s-LNvs are important to circadian timekeeping, recent work has shown that abrogating the s-LNvs does not lead to significant circadian changes in the insect, nor the functional output of s-LNv neurons. Instead, there is a reduction in glutamate sensory neurotransmission, which in turn disrupts the integration of environmental time cues (Song et al., 2021). This suggests the neural plasticity in this clock mediated LNV-DN1a network is critical for integrating sensory inputs from light to gate circadian entrainment.

### Circadian rhythms affect cognition in mice

Though studying synaptic plasticity of the clock is more difficult in mouse models than *Drosophila*, due to the relatively defined number of clock neurons in *Drosophila*, there is substantial evidence that the circadian clock plays a strong role in learning and memory in rodent models. This section will focus on the mechanisms by which short and long term memory as well as long term potentiation (LTP) are thought to be under control of the circadian clock system. It is well-known that learning and memory are modulated throughout the day. For example, rats trained to avoid a foot shock by staying in one area of their chamber (known as a passive avoidance task) display better learning and recall when trained during the active phase versus the rest phase (Davies et al., 1973). This time of day (TOD) effect on learning and memory is present in many model organisms (Gerstner et al., 2009). Within mouse models, global knockout of the circadian clock *via* deletion of *Bmal1* elicits substantial body wide changes including deficits in learning, memory and mood (Price and Obrietan, 2018). Because body wide loss of BMAL1 results in poor health and severely accelerated aging (Kondratov et al., 2006), multiple tissue-specific knockout models have been created. For example, nestin-driven brain-specific *Bmal1* knockout mice show hyperactivity, synaptic degeneration and astrogliosis (Musiek et al., 2013). Conditional knockout of *Bmal1* only in the prefrontal neurons (leaving the SCN clock fully functional) results in poor performance in TOD-dependent learning and memory tests like novel object recognition and the Barnes Maze tests. This particular mouse model indicates that forebrain excitatory neurons are critical for the circadian effects on learning and memory. (Snider et al., 2016; Price and Obrietan, 2018).

While circadian clock genes are known to be important for normal learning and memory, some intracellular signaling pathways important for neuronal function are thought to be important in learning and memory based on their rhythmic activation. For example, the mitogen activated protein kinase interacting kinase (MNK)-eukaryotic translation initiation factor-4E eIF4E pathway has previously been shown to regulate mRNA translation and modulates SCN function (Liu et al., 2022). Phosphorylation of eIF4E occurs in a diurnal fashion throughout neurons. Mice in which eIF4E phosphorylation is prevented by mutation of MNKs (which phosphorylate eIF4E), show impaired diurnal variation of cognitive functions like memory and learning (Liu et al., 2022).

While the ERK/MAPK pathway is light-driven in the SCN, SCN rhythmicity is necessary for rhythmic ERK/MAPK activity in the hippocampus. Both global *Bmal1* KO mice and mice with lesioned SCNs lack diurnal hippocampal ERK phosphorylation (Phan et al., 2011). It is has been demonstrated that the SCN-derived ERK/MAPK inhibitor, suprachiasmatic nucleus circadian oscillatory protein (SCOP), regulate in part these hippocampal rhythms in ERK activity. Nucleotide-free Ras protein binds to GTP to activate the ERK/MAPK cascade; however, when SCOP is present, it sequesters Ras. Typically SCOP is degraded when neural firing leads to Ca2 + influx activating calpain, a calcium dependent protease which degrades SCOP (Shimizu et al., 2007). During the nighttime, SCOP localized to the membrane raft, is at its zenith. Interestingly, TOD-dependent hippocampal ERK activation is absent in SCOP knockout mice (Shimizu et al., 2016). Thus, the production of SCOP by the SCN is likely important for ERK-mediated activation leading to circadian effects on learning and memory.

An essential cue for the brain’s clock is the production and secretion of the stress hormone cortisol, either in response to or in anticipation of internal or external cues. Under prolonged stress, CRH neurons increase production of cortisol. Long-term elevations in cortisol are linked to synaptic loss and branch atrophy in cortical and subcortical areas, ultimately leading to problems in cognition (Popoli et al., 2011; Csabai et al., 2018). In mice exposed to aberrant light cycles, depressive-like behaviors have been demonstrated, as well as a rise in serum corticosterone levels (LeGates et al., 2012). Moreover, hippocampal learning and memory, as assessed by the Morris Water Maze and novel object recognition tasks are also impaired by altered exposure to the light/dark cycle. Glucocorticoid rhythms promote rapid post-synaptic dendritic spine growth after motor skill learning, with decreased amounts of glucocorticoids needed to stabilize newly formed spines and thus enable long-term memory formation (Liston et al., 2013). In contrast, the mineralocorticoid receptor activation pathway can suppress spine formation.

### Circadian rhythms, plasticity, and sleep

While homeostatic mechanisms are partly responsible for regulation of sleep, circadian rhythms in the brain also play an integral role. Recently, the “State Clock” model has been proposed, which suggests that many of the synaptic changes driven by the sleep-wake cycle are in fact driven by the circadian clock (Frank and Cantera, 2014). The role of sleep in plasticity is multifaceted. For example, the fruit fly has increased motor neuron synapses during sleep, while also having decreased axonal processes of mushroom body gamma neurons following sleep (Weiss and Donlea, 2021). In mice, sleep deprivation results in robust transcriptional and translational changes, revealing that multiple biological pathways are ultimately affected. One such manifestation can be seen in the somatosensory cortex, which has more excitatory synapses during the daytime and increased inhibitory synapses during the nighttime (Jasinska et al., 2014, 2015). Thus, similar to flies, the timing of sensory inputs may regulate the mouse’s synaptic density in the somatosensory cortex.

Melatonin, which is integral to the sleep-wake cycle, is also thought to have a role in the timing of hippocampus-mediated learning. Melatonin receptors are present in the hippocampus and when the melatonin 2 receptors are knocked out in mice, there is impairment of long-term potentiation, a plasticity thought to serve as the foundation for long term memory formation (Larson et al., 2006). Furthermore, C57/BL6 mice, which are deficient in melatonin, treated with melatonin in the evening display better working memory performance in the daytime (Jilg et al., 2019). Mice lacking melatonin receptors or mice with surgically removed pineal glands, were shown to score lower in spatial learning tests, regardless of daytime or nighttime. This suggests melatonin receptor signaling is responsible for the improved learning seen during the daytime in mice.

Other ways to study the relationship of sleep and circadian rhythms in plasticity is by disrupting rhythms. For example, to model the effects of shift work schedules, rodents can be placed in a simulated shift work schedule using forced activity during their rest phases to simulate the night shift (Marti et al., 2020). Interestingly, rats subjected to shift work followed by cognitive testing showed substantially impaired spatial memory. Subsequent analysis of the brain tissue from these rats reflected altered clock-driven protein expression in the prefrontal cortex along with altered corticosterone levels. These preclinical studies may highlight some of the underlying mechanisms leading to reduced cognitive performance of humans under shift work (Rouch et al., 2005; Devore et al., 2013). In developing adolescents, consistent long-term disruption of circadian rhythms, often *via* disruption of the sleep/wake cycle is associated with an increased predisposition for addiction and substance abuse in adolescents (Logan et al., 2018). It is thought that changes in the sleep/wake cycle may impact the risk/reward balance in adolescents. This is further supported by recent findings wherein researchers compared transcriptional rhythms in the brains of patients with and without opioid use disorder. Using time of death as a marker of time of day, this study revealed fewer rhythmic transcripts in the dorsolateral prefrontal cortex of deceased individuals with opioid use disorder compared to control individuals, while in the nucleus accumbens (NAc) the number of rhythmic transcripts was nearly double compared to controls (Xue et al., 2022). Furthermore, analysis of the NAc revealed that transcripts heavily involved in dopamine (DA), GABAergic, and glutamatergic synaptic functions were altered between control and opioid use disorder individuals. Lastly, the rhythmic transcripts found in the NAc and the dorsolateral prefrontal cortex of patients with opioid use disorder were enriched for genomic loci associated with sleep-related issues (Xue et al., 2022). In summary, studies in humans are beginning to more fully reveal potential mechanisms involved in the relationships between sleep, circadian rhythms, and neural plasticity.

### Circadian rhythms and mood

Psychiatric disorders are linked to the circadian clock. In the late 1980s, the “Social *Zeitgeber* Theory” postulated that in some individuals with predisposed risk for mood-related disorders, stressful life events lead to changes in the sleep-wake cycle, which alters molecular and cellular rhythms resulting in an increased risk for mood-related disorders (Ehlers et al., 1988). Growing support for this theory has been generated in several clinical and preclinical studies, which have underscored a connection between chronodisruption and mood or behavioral changes (Germain and Kupfer, 2008; Howland, 2009; McClung, 2013; Landgraf et al., 2014b). Genetic screening of individuals with psychiatric illness reveal a common occurrence of polymorphisms in 21 circadian clock genes (Etain et al., 2014). Furthermore, preclinical evidence of a clock regulated aspect to psychiatric illnesses is the clock delta 19 mutant mouse model, an arrhythmic mouse, which displays mania-like behavior (Roybal et al., 2007). One human study addressing circadian rhythms in depressed individuals monitored body temperature, cortisol, norepinephrine, thyroid stimulating hormone, and melatonin, comparing depressed individuals to “recovered” individuals treated with anti-depressants or normal subjects. Compared to recovered and normal subjects, rhythms of depressed individuals showed reduced amplitude; however, following recovery their rhythms return to normal amplitude (Souêtre et al., 1989). Many current treatments for mood disorders aid in shifting or stabilizing circadian rhythms of the individual (Kripke et al., 1978; Gottlieb et al., 2021; Silva et al., 2021). However, it is often difficult to distinguish whether the disruption of the circadian clock is a result of the changes in mood or rather the initiator of the mood disorder (Silva et al., 2021). The evidence presented here further supports the circadian system as an active participant in crucial brain functions to regulate mood and behavior *via* plasticity.

Aberrant synaptic plasticity often characterizes psychiatric illnesses. One hypothesis is that neurotransmission is impaired in psychiatric illness leading to synaptic remodeling. Decreased DA levels are associated with depression, while increased levels appear to contribute to mania (Berk et al., 2007). DA has 24-h oscillations in the striatum and NAc that are reduced by light (Castañeda et al., 2004). *In vivo* recording of dopaminergic neurons in clock delta 19 mutant mice, which display mania like behavior, have revealed increased firing and bursting of the dopaminergic neurons in the ventral tegmental area (VTA) (McClung et al., 2005). When CLOCK is expressed only in the VTA of clock mutant mice, the mania-like behavior is substantially diminished, indicating that CLOCK protein may mediate mania-like behavior, potentially by affecting neurotransmitters like DA (Roybal et al., 2007). Interestingly, hypothalamic expression of DA in rats can actually be switched to the somatostatin neurotransmitter by mimicking a summer versus winter day length (Dulcis et al., 2013). Following the switch to somatostatin, rats also display parallel changes in the post-synaptic expression and increased anxiety behaviors (Dulcis et al., 2013). Altogether it is likely through a mixture of changes in synaptic plasticity and neurotransmission that our internal clocks can mediate our mood (McClung, 2013; Bechtel, 2015).

## Circadian rhythms in the central nervous system during aging

It is well known that our normally robust circadian cycle loses robustness during aging. A good example of this is the fragmentation of the sleep/wake cycle commonly seen in elderly adults. Dampened rhythms in physiology and behavior are supported by molecular observations at the cell and tissue levels. Multiple metabolic pathways supporting neuronal activity are dampened with age, and may precipitate aging-related cognitive decline.

One such pathway which decays over time is the SIRT1 pathway in the SCN. One study by Chang et al. (Chang and Guarente, 2013) showed that SIRT1, the NAD^+^-dependent protein deacetylase, participates in a circadian amplifying loop with PGC-1alpha and *Nampt* to regulate *Bmal1* transcription. In aged mice, SIRT1 expression is highly reduced along with other circadian molecules, leading to disruptive activity and a reduced capacity to adapt to changes in the light cycle. Young mice lacking SIRT1 expression display similar circadian issues as the aged mice. Moreover, when SIRT1 was overexpressed in the brains of aged mice, the aged mice were protected from the age associated circadian issues (Chang and Guarente, 2013). Thus, SIRT1 likely contributes to SCN mediated rhythms which decline over time.

Multiple studies have validated the diurnal variation in the onset of cerebrovascular disease [reviewed in Fodor et al. (2021)]. Interestingly, there is a significantly higher onset of ischemic stroke (IS) onset in the morning compared to other times of the day. The reasons for this are complex, but due to the fact that normotensive and hypertensive individuals have similar circadian rhythms in IS onset, the diurnal incidence is thought to be attributable at least in part to circadian variations in blood pressure. The diurnal variance is also thought to be potentially dependent on hypercoagulability in the morning compared to other circadian times (Kapiotis et al., 1997). While blood viscosity is highest in the morning, tissue plasminogen activator (tPA) activity as at its circadian nadir (Kurnik, 1995).

### Aging associated diseases of the central nervous system

In aging-associated diseases of the brain, such as Alzheimer’s, the sleep/wake cycle is highly impaired. Interestingly, new evidence suggests that the circadian clock may be heavily involved in this effect of AD on sleep (Chauhan et al., 2017; Musiek et al., 2018; Hablitz et al., 2020; Clark et al., 2022; Hoyt and Obrietan, 2022). For example, a recent study suggests that turnover of the cerebrospinal fluid occurs in a circadian fashion (Hablitz et al., 2020). Furthermore, phagocytosis of amyloid beta precursors is time-of-day dependent (Clark et al., 2022). Thus the processes that may prevent the onset or progression of AD may be under circadian control. Modeling sleep-dependent clearance of waste in the brain predicts timescales of tau accumulation that are consistent with the development of Alzheimer’s disease (Tekieh et al., 2022). The circadian regulation of glymphatics in AD progression is an exciting area that will undoubtedly assist in our understanding of the links between aging and cognitive decline. Changes in memory vary drastically as between individuals during aging. Some of this variation in memory loss as we age has a circadian component. Multiple studies have shown that fear conditioning memory is more efficient if carried out during the daytime than the nighttime (Chaudhury and Colwell, 2002; Eckel-Mahan et al., 2008). Long term potentiation (LTP) is key to memory formation and is regulated in a circadian fashion in the hippocampus (Chaudhury et al., 2005). Research has begun to make headway in understanding the circadian role in the synaptic plasticity of long term potentiation (LTP). Using electrophysiological hippocampal slice recording, disinhibition of hippocampal neurons by recurrent inhibitory GABAergic input has been shown to modulate LTP (Nakatsuka and Natsume, 2014). The molecular pathway of LTP is regulated by activation of the cyclic adenosine monophosphate response element (CREB) though MAPK and cAMP pathways. In mice, the hippocampus undergoes oscillatory changes in cAMP and CREB phosphorylation throughout the day. Interestingly, in the absence of PER1 protein, memory-induced CREB phosphorylation, which normally occurs during the daytime, is completely absent (Rawashdeh et al., 2016). Furthermore, in WT mice, the CREB kinase pP90RSK interacts with PER1 to mediate its translocation into the nucleus. When PER1 expression is abolished, pP90RSK does not undergo nuclear translocation, preventing CREB phosphorylation and activation of gene transcription. Thus PER1 interacts with the CREB kinase pP90RSK to gate CREB activation in learning and memory (Rawashdeh et al., 2016). In aged mice, these rhythms are often dampened and the mice display reduced memory performance suggesting a circadian mediated role in memory formation that is reduced in the aging brain.

An important protein linking circadian rhythms and neuronal function is glycogen synthase kinase beta 3 (GSK3), whose diurnal activity regulates hippocampal clock gene expression and plasticity and is correlated with enhanced nighttime LTP (Besing et al., 2017). It is not fully understood how GSK3β may mediate LTP, however, one can posit that it may be impart *via* its ability to promote membrane localization of NMDA receptors (Chen et al., 2007). When consistently activated in a GSK3β knock in mouse, diurnal LTP is still observed suggesting the GSK3β pathway is not the only one involved in temporally gating LTP. ERK, which is another kinase under circadian control in the hippocampus, is also involved in LTP. ERK can regulate potassium channels to increase neuronal excitability in the hippocampus (Impey et al., 1998). Both GSK3β and ERK are circadian regulated kinases capable of altering the hippocampal landscape and implicated in age-related memory loss.

In fruit flies, aging is also associated with a decline in circadian rhythms of activity and the PDF hormone. Specifically, one study compared 1−, 30−, 40−, and 50-day old flies and revealed that there was an age-associated decline in PDF (Umezaki et al., 2012). Additionally, the anticipatory activity to the light dark cycle is reduced throughout aging, based on monitoring fly locomotion (Curran et al., 2019). s-LNv PDF terminal remodeling is reduced in 30 day old flies. In addition, while whole cell patch clamp electrophysiology recording from young and aged I-LNv neurons revealed no major difference between spontaneous activities, input resistance was reduced as an effect of age. Interestingly, in mice, the input resistance is not majorly affected across age; however, *in vivo* electrical activity of mouse neurons, and in organotypic slices the day-night firing rates vary diurnally (Nakamura et al., 2011; Farajnia et al., 2012). It is important to note that melatonin, the rhythms of which decrease in amplitude in aged individuals, is tied to time of day dependent LTP (Jilg et al., 2019). Comparing WT mice to melatonin knockout mice, reveals that day/night synaptic weight differences are missing in melatonin-deficient mice along with altered CREB rhythms. Thus, melatonin may mediate synaptic weights in a diurnal fashion, which entrains memory formation (Jilg et al., 2019).

Some studies suggest that there may be roles for circadian clock genes in memory that extend beyond their participation in the 24-h transcriptional/translational feedback loop of the core clock (Kwapis et al., 2018). For example, Kwapis et al. (2018) demonstrate that the repressive histone deacetylase (HDAC3), which alters gene expression *via* epigenetic mechanisms, contributes to age-related memory impairment by inhibiting *Per1* expression and acetylation, while not affecting rhythmicity of the SCN or the organism. Interestingly, *Per1* overexpression in the dorsal hippocampus was sufficient to restore aging-associated deficits in object location memory. Though not directly shown in this study, since PER1 is upstream of CREB phosphorylation (Rawashdeh et al., 2016), it may be that age-associated decline in PER1 abundance limits CREB-driven gene expression in the hippocampus.

### Exercise and the plasticity in the aging brain

Increasing evidence points to a protective effect of exercise on neuronal activity throughout aging. Like light and energy intake, physical activity affects both sleep and circadian rhythms. Timed daily exercise has been shown to act as a *zeitgeber*, which can reset and remodel circadian rhythms in mice (Choi et al., 2020; Hughes et al., 2021). Evidence points to exercise-induced alterations in peripheral rhythms as important for sleep modulation. For example, tissue specific rescue of *Bmal1* expression in skeletal muscle of whole body *Bmal1*-knockout can alter the sleep-wake cycle, and overexpression of BMAL1 in skeletal muscle can reduce the recovery response to sleep loss in mice (Ehlen et al., 2017). In mice, even the introduction of a running wheel in aged mice not previously exposed to wheel running, can lead to changes toward a younger brain phenotype (Panagiotou et al., 2018). Even moderate amounts of exercise have been demonstrated to improve cognitive functions in elderly individuals (Carta et al., 2021). Sleep studies looking at sleeping patterns in older men before and after chronic exercise, shows that sleep quality improves following exercise (Melancon et al., 2015). The benefits of exercise on the brain are not fully understood; however, some studies link the hormone BDNF and to changes in plasticity in areas like the hippocampus (Aguiar et al., 2011). The circadian regulated hormone Brain-derived neurotrophic factor (BDNF) is critical in synaptic formation for shaping learning and memory (Schaaf et al., 2000; Messaoudi et al., 2002; Kowiański et al., 2018). Exercise induces BDNF production in the brain, which corresponds with improved cognitive performance and mood (Vaynman et al., 2004a,b). While BDNF has numerous mechanisms of action, it promotes TrkB-mediated induction of glutamate receptor subunit expression and synaptic delivery of AMPA receptors in the hippocampus (Caldeira et al., 2007; Fortin et al., 2012). This indicates BDNF has an ability to modulate local plasticity at synapses. In addition, *via* interaction with a truncated TrkB.T1, BDNF stimulates *Nrf2* transcription in astrocytes during the rest phase. Production of Nrf2, a redox sensitive transcription factor, aids in protecting astrocytes from the buildup of reactive oxygen species during the rest phase (Ishii et al., 2019). The BDNF-TRKB pathway also activates clock-regulated mammalian target of rapamycin complex (mTOR1), which acts as a neuronal response regulator by modulating production of proteins necessary for spines and synapses (Henry et al., 2012; McCabe et al., 2020). Interestingly, N-acetyl serotonin can also activate the TrkB receptor, increasing its activity at night compared to the day (Jang et al., 2010). Even short bouts of exercise in rats is sufficient to stimulate an increased production of BDNF, which is accompanied by elevated cAMP and CREB in the hippocampus of aged rats (Aguiar et al., 2011). Thus, BDNF and its signaling cascade contribute to rhythmic activity *in vivo*. Collectively, these studies support the idea that exercise is a way to slow cognitive decline throughout aging. Whether the entrainment effects of exercise shape plasticity in the brain preventing age-associated cognitive decline, or whether exercise works *via* other more important non-timing dependent pathways to ward off the physiological wear of time is not fully clear. However, it is clear that diurnal exercise offers a solution to improving circadian rhythms and slowing age-related cognitive decline.

## Conclusions and future directions

In conclusion, regulation of the nervous system by the circadian system occurs early in development and extends throughout life. While early disruption of rhythms can lead to cognitive and behavioral defects later in development, aging appears to also promote cognitive decline by dampening clock function. Thus, preserving a robust internal clock may one of the most important mechanisms by which to delay age-associated cognitive decline.

Recently, numerous pharmacological and behavioral manipulations have been tested, largely using preclinical models, to determine whether augmenting clock function can increase circadian robustness throughout aging and prevent cognitive decline (He et al., 2016; Kim et al., 2021; Wirianto et al., 2022). For example, studies using a natural flavonoid Nobiletin (NOB), which is also a modulator of the circadian clock, reveal that augmenting clock function may be a mechanism by which to delay cognitive decline throughout aging and particularly, in the context of AD. NOB is a natural polymethoxylated flavone, which functions as a clock amplitude-enhancing small molecule. In the context of diet-induced obesity, it can mitigate metabolic effects associated with diet-induced obesity in a *Clock*-dependent manner (He et al., 2016). Interestingly, NOB has been shown to affect astrogliosis in a mouse model of AD, and NOB was also shown to reduce the expression of several AD-driving genes in female AD mice, in particular (Kim et al., 2021; Wirianto et al., 2022). NOB works predominantly as a retinoic acid receptor-related orphan receptor (ROR) agonist, and has been shown to have anti-tumor effects in the context of triple negative breast cancer, where it alters NF-κB activity in a ROR-dependent manner (Kim et al., 2022). More studies will be needed to determine the extent to which the preclinical studies showing NOB effects on AD-related genes may be extended to the human brain throughout aging and disease.

Light, which plays an important role in the relationship between circadian rhythms in the brain and mood, offers a potential therapeutic opportunity for individuals with circadian related disruption. Most notably, seasonal affective disorder (SAD), characterized by seasonal depression during the onset of winter when environmental light is limited compared to other seasons, is a prime example of how light entrainment can alter mood. The treatment for SAD was first tested on seasonally manic depressive patients who were given 3 h of artificial bright light exposure upon awakening (Rosenthal et al., 1984). The light treatment greatly ameliorated their depressive symptoms. However, when the treatment was stopped the patients depressive symptoms returned, indicating the immediate and temporary affect light entrainment played in subjects’ mood. While light has ameliorating effects when applied during the daytime in humans, chronic light at night can induce depressive symptoms (Bedrosian and Nelson, 2013; Obayashi et al., 2013, 2014). Viral tracing studies have revealed a pathway from the light-sensitive retinal ganglion cells (IpRGCs) to the dorsal habenular nucleus (dpHb) to the NAc that mediates light at night-induced depression (An et al., 2020). These findings highlight light therapy as a powerful non-invasive therapeutic for individuals suffering from circadian related mental health problems.

In conjunction with light therapy the use of melatonin supplements can be an effective mechanism to phase shift the circadian clock. In elderly individuals who are more prone to circadian misalignment, melatonin production is greatly reduced. Controlled release of melatonin offers a method to improve sleep quality in the elderly with low side effects (Garfinkel et al., 1995). Additionally, in some blind individuals not entrained to the light/dark cycle, melatonin supplementation can assist with entrainment (Lockley et al., 2000). In the context of travel across time zones or even social jet lag, melatonin can function as both a preventative treatment or a ameliorative treatment for circadian misliagnment (Herxheimer and Petrie, 2001). Although melatonin can be used as chronotherapy, more research is needed to fully understand why some individuals are more responsive to others in behavioral response. While light and melatonin are commonly used to treat circadian misalignment, another behavioral method used to reinforce the internal biological clock is time-restricted feeding, which can dramatically increase robustness of the peripheral clocks, delay diet-induced metabolic disease, and promote healthy aging (Hatori et al., 2012; Chaix et al., 2014, 2019; Acosta-Rodríguez et al., 2022). It remains to be determined whether implementing such approaches earlier during development would improve cognition throughout aging.

In summary, preservation of our internal biological clock at all stages of life appears to be critical for optimal brain development, sleep, and cognition. A deeper understanding of the dynamic 24-h function within the central nervous system at all stages of development promises to be important for promoting maximal central nervous system function throughout aging and potentially, chronotherapy for a variety of mental illnesses.

## Author contributions

RVD and KE-M both contributed to the writing of the manuscript and jointly approved the submitted version.
